# Antioxidant Potential of Diosmin and Diosmetin against Oxidative Stress in Endothelial Cells

**DOI:** 10.3390/molecules27238232

**Published:** 2022-11-25

**Authors:** Magdalena Wójciak, Marcin Feldo, Grzegorz Borowski, Tomasz Kubrak, Bartosz J. Płachno, Ireneusz Sowa

**Affiliations:** 1 Department of Analytical Chemistry, Medical University of Lublin, Chodzki 4A, 20-093 Lublin, Poland; 2 Department of Vascular Surgery, Medical University of Lublin, Staszica 11 St., 20-081 Lublin, Poland; 3 Department of Biochemistry and General Chemistry, Medical College of The University of Rzeszów, 2A Kopisto St., 35-959 Rzeszów, Poland; 4 Department of Plant Cytology and Embryology, Institute of Botany, Faculty of Biology, Jagiellonian University in Kraków, Gronostajowa 9 St., 30-387 Cracow, Poland

**Keywords:** H_2_O_2_ stress, diosmin, diosmetin, endothelial cells, chronic venous insufficiency

## Abstract

Phlebotropic flavonoids, including diosmin and its aglycone diosmetin, are natural polyphenols widely used in the prevention and treatment of chronic venous insufficiency (CVI). As oxidative stress plays an important role in the development of pathophysiology of the cardiovascular system, the study aimed to investigate the protective effects of diosmin and diosmetin on hydrogen peroxide (H_2_O_2_)-induced oxidative stress in endothelial cells. The cells were pretreated with different concentrations of the flavonoid prior to the H_2_O_2_ exposure. The cell viability, the level of intracellular reactive oxygen species (ROS), the activity of cellular antioxidant enzymes—including superoxide dismutase (SOD), catalase (CAT), and glutathione peroxidase GPx—and the malondialdehyde (MDA) level were assessed. It was found that the H_2_O_2_-induced oxidative stress was ameliorated by diosmin/diosmetin in a concentration-dependent manner. The flavonoids restored the activity of cellular antioxidant enzymes and lowered the MDA level upregulated by the H_2_O_2_ exposure. These results indicate that diosmin and diosmetin may prevent oxidative stress in endothelial cells; therefore, they may protect against the development and progression of oxidative-stress-related disorders.

## 1. Introduction

Oxidative stress associated with the excessive production of reactive oxygen species (ROS) favors the damage and dysfunction of cells and tissues and negatively affects physiological processes in many organs. ROS contribute to damage to nucleic acids and lipids, alter the structure and/or function of proteins, and thus promote structural changes in tissues. Moreover, ROS alter some cellular signaling pathways via interaction with critical signaling molecules, thereby affecting various cellular processes, such as proliferation, migration, differentiation, and apoptosis of cells. ROS also influence the extracellular matrix components, including matrix metalloproteinase (MMPs), which are responsible for tissue remodeling [1,2].

It has been evidenced that oxidative stress caused by redox imbalance is involved in pathological processes in the organism and participates in the development of various diseases, including cardiovascular disorders (CVD) [2]. Many reports suggest an important role of endothelial dysfunction in CVD progression; therefore, protection of the endothelium against ROS may alleviate CVD symptoms [3,4,5,6]. The endothelial layer is responsible for proper vascular tone, vessel wall permeability, and functioning of the coagulation/fibrinolytic system. Its dysfunction promotes unfavorable processes in blood vessels, including adhesion and migration of leukocytes, aggregation and adhesion of platelets, thrombosis, and increased vascular tone [7,8,9,10]. The endothelium maintains the local balance between pro- and anti-inflammatory factors, and its dysfunction leads to local changes in the vessels and disturbance of homeostasis [3,11]. Additionally, the impairment of the synthesis of endothelium-derived NO associated with endothelial dysfunction results in or enhances multiple adverse effects, including vasoconstriction and thrombosis, as well as the expression of proinflammatory cytokines and chemokines [3,6].

Recent reports considered antioxidants, including polyphenols, as useful factors preventing the development and progression of disorders related to oxidative stress [12,13]. Diosmin and its aglycone diosmetin are natural polyphenolic compounds commonly found in pericarps of different citrus fruits (Rutaceae). They are known for their effectiveness in supporting the treatment of chronic venous insufficiency (CVI) belonging to a group of cardiovascular disorders. Many clinical studies proved that diosmin alleviates CVI symptoms, including inflammation, edema, skin lesions, swollen legs, and structural changes in the vein wall, such as varicose veins and venous leg ulcers [7,8,9,10]. Furthermore, it has a protective effect on blood vessels, increases the tension and elasticity of vessel walls, and reduces the permeability of capillary walls [14,15,16].

Some clinical and in vivo studies have evidenced the protective efficacy of diosmin/diosmetin against CVI symptoms at the molecular level [7,8,9,10]. It was found that diosmin administration in patients with CVI decreased the levels of selected proangiogenic factors involved in CVI pathology and reduced pro-inflammatory cytokines, including TNF and IL-6 [10,17]. Moreover, it decreased the concentration of isoprostanes, which are the main product of nonenzymatic lipid peroxidation of polyunsaturated fatty acids (PUFA) and are considered a marker of oxidative stress [17]. Our previous research showed a modulatory effect of diosmin and diosmetin on proinflammatory factors in human skin fibroblasts stimulated with lipopolysaccharide and their strong inhibitory activity on elastase and collagenase [18]. In addition to CVI, diosmin exerts beneficial activity in hemorrhoidal disease, improves cardiac function, and has anti-inflammatory, antinociceptive, antifibrotic, antidiabetic, and antihyperlipidemic properties [19].

Although some studies describe protective effects of diosmin/diosmetin against various inducers in animal models or on different types of cells [19,20,21], there are no reports on their protective effects against oxidative stress in the endothelium.

Therefore, the aim of the present study was to investigate the role of these flavonoids in protection of the endothelial layer against oxidative damage caused by H_2_O_2_ exposure as a model of oxidative stress. The impact of the pretreatment with the flavonoids on the level of intracellular reactive oxygen species (ROS), the activity of antioxidant enzymes, including SOD, CAT, and GPx, and the level of malondialdehyde (MDA) regarded as an indicator of lipid peroxidation was evaluated. The NO concentration was also assessed because it plays a pivotal role in the regulation of endothelial function.

## 2. Results

### 2.1. Cytotoxicity Assay

To establish the nontoxic concentration of diosmin and diosmetin, the cytotoxicity of the flavonoids towards endothelial cells was assessed using MTT and Alamar Blue (AB) assays. It was noted that, up to 250 µM, diosmetin did not affect the cellular metabolism, the stability of cell membranes and the cell morphology. In turn, diosmin at a concentration of 250 µM and diosmetin at a concentration of 300 µM decreased cell viability (to 90% and 84%, respectively, compared to the control) and the mitochondrial dehydrogenase level at 300 µM decrease cell viability to 86% and 82%, respectively (Supplementary Materials Figure S1). Their cytotoxicity was enhanced at higher concentrations. Therefore, the concentrations up to 250 µM were used in further investigations.

**Figure 1:**
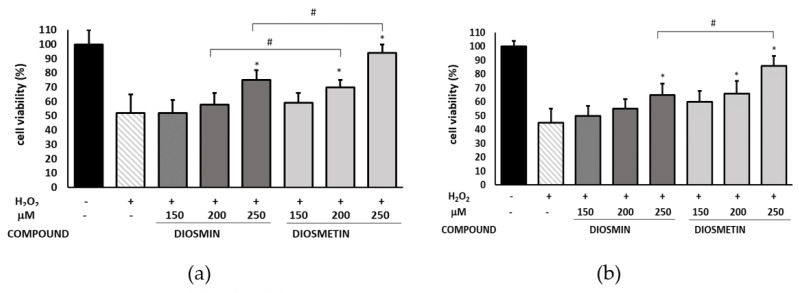
Cell viability determined by the MTT (**a**) and Alamar Blue (**b**) assays expressed as a percentage of the control (0.5% DMSO). Cells were treated with H_2_O_2_ or pretreated with diosmin/diosmetin at different concentrations prior to the H_2_O_2_ exposure. The data are means ± SD (*n* = 3). * indicates a statistically significant difference (*p* < 0.05) versus H_2_O_2_-treated cells assessed using one-way ANOVA followed by Dunnett’s multiple comparison post hoc test; ^#^ indicates a statistically significant difference (*p* < 0.05) between the same concentrations of diosmin and diosmetin assessed using Student’s *t*-test.

To evaluate the protective effect of diosmin/diosmetin, endothelial cells were preincubated with the flavonoids prior to H_2_O_2_ exposure. It was found that the H_2_O_2_ stimulation had a negative impact on cell viability and decreased the percentage of viable cells established using the MTT and Alamar Blue assays to 52% and 45%, respectively. In turn, the preincubation with the flavonoids attenuated the H_2_O_2_-induced cytotoxicity in a concentration-dependent manner (Figure 1 and Supplementary Materials Figure S2). Moreover, diosmetin was found to act more effectively than diosmin: at the concentration of 250 µM, the percentage of viable cells was approx. 19% (MTT) and approx. 21% (AB) higher than when diosmin was used.

A further assay aimed to find out whether the protective effects of the flavonoids on cell viability after the H_2_O_2_ exposure resulted from their impact on oxidative imbalance.

### 2.2. Antioxidant Activity

To evaluate the protective effect of diosmin/diosmetin against disturbance in the oxidative balance, the influence on the intracellular production of reactive oxygen species and enzymatic activity of cellular antioxidant systems involved in protection against ROS was determined.

#### 2.2.1. Level of Intracellular Reactive Oxygen Species

**Figure 2:**
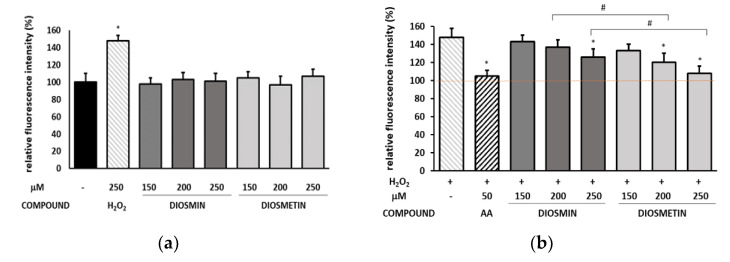
Relative fluorescence intensity in endothelial cells calculated as a percentage in comparison with untreated control cells. (**a**)—the cells were treated with H_2_O_2_ or different concentrations of diosmin/diosmetin, * indicates a statistically significant difference (*p* < 0.05) versus untreated controls; (**b**)—the cells were pretreated with diosmin or diosmetin prior to the H_2_O_2_ exposure. The data are means ± SD (*n* = 3). * indicates a statistically significant difference (*p* < 0.05) versus the H_2_O_2_-treated cells. One-way ANOVA followed by Dunnett’s multiple comparison post hoc test. ^#^ indicates a statistically significant difference (*p* < 0.05) between the same concentrations of diosmin and diosmetin assessed using Student’s *t*-test. AA—ascorbic acid. The red dashed line represents the control value that was considered as 100%.

The H_2_DCFDA test was used to determine the ability of the flavonoids to reduce the intracellular production of reactive oxygen species in endothelial cells induced with H_2_O_2_. The results are shown in Figure 2 and Supplementary Materials (Figure S3).

As can be seen, the flavonoids did not affect the oxidative balance, and H_2_O_2_ significantly increased the ROS level up to 148% compared to the control (Figure 2a). However, the pretreatment with the flavonoids lowered ROS production in a concentration-dependent manner. Diosmetin exerted a higher protective effect, as at the highest concentration, the amount of ROS was almost restored to the level of the untreated control.

#### 2.2.2. Enzymatic Activity

**Figure 3:**
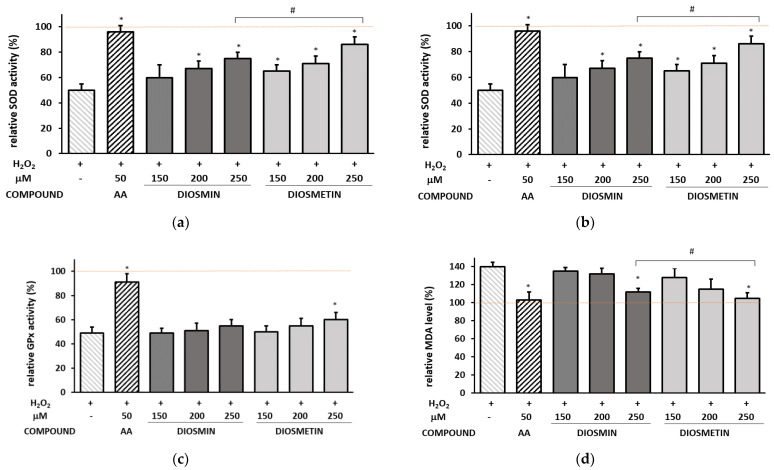
Effect of the diosmin/diosmetin pretreatment prior to the H_2_O_2_ exposure on the antioxidant enzyme activity calculated as a percentage in comparison with the untreated control. (**a**)—relative activity of superoxide dismutase (SOD), (**b**)—relative activity of catalase (CAT) (**c**)—relative activity of glutathione peroxidase (GPx) (**d**)—relative malondialdehyde (MDA) concentration. The data are means ± SD (*n* = 3). * indicates a statistically significant difference (*p* < 0.05) versus the H_2_O_2_-treated cells. One-way ANOVA followed by Dunnett’s multiple comparison post hoc test. ^#^ indicates a statistically significant difference (*p* < 0.05) between the same concentrations of diosmin and diosmetin assessed using Student’s *t*-test. AA—ascorbic acid. The red dashed line represents the control value that was considered as 100%.

Since our previous study showed a low direct ability of diosmin and diosmetin to scavenge free radicals [18], at this stage of the research, the effect of the flavonoids on the activity of cellular antioxidant enzymes, such as SOD, CAT, and GPx, was investigated. Moreover, the effect of the flavonoids on lipid peroxidation was assessed using malondialdehyde (MDA) as the main end product of lipid peroxidation. The results obtained for cells without the H_2_O_2_-induced oxidative stress and for cells pretreated with the flavonoids prior to the H_2_O_2_ exposure are shown in Figure 3 and Supplementary Materials (Figure S4), respectively.

It was found that diosmin and diosmetin at 200–250 µM significantly affected the SOD and CAT activity in H_2_O_2_-exposed cells; however, they had a minor impact on GPx—only the highest concentration of diosmetin slightly increased GPx compared to the H_2_O_2_-induced cells. Moreover, it was noted that diosmin and diosmetin at 250 µM decreased the MDA level almost to that in the control cells.

### 2.3. Modulation of the Nitric Oxide (NO) Level

**Figure 4:**
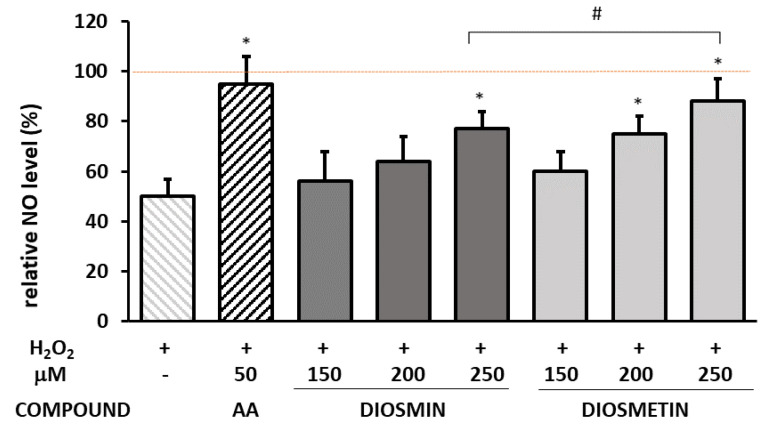
Effect of diosmin/diosmetin on the NO level calculated as a percentage in comparison with the untreated control (100%). The data are means ± SD (*n* = 3). * indicates a statistically significant difference (*p* < 0.05) versus the H_2_O_2_-treated cells assessed using one-way ANOVA followed by Dunnett’s multiple comparison post hoc test. ^#^ indicates a statistically significant difference (*p* < 0.05) between the same concentrations of diosmin and diosmetin assessed using Student’s *t*-test. AA—ascorbic acid. The red dashed line represents the control value that was considered as 100%.

The intracellular nitric oxide was measured in endothelial cells treated with H_2_O_2_ or diosmin and diosmetin at different concentrations (Supplementary Materials Figure S5) and in cells pretreated with flavonoids prior to the H_2_O_2_ exposure (Figure 4).

The H_2_O_2_ exposure inhibited the NO production, and the NO level was significantly decreased (to 56% of the control). The NO level was also slightly reduced in the flavonoid-treated cells (Supplementary Materials Figure S3). In cells preincubated with flavonoids prior to oxidative stress induction, NO was higher compared to H_2_O_2_-treated cells and, at the highest concentration of the flavonoids, it reached the level of 77% (diosmin) and 88% (diosmetin) of that in the untreated control cells.

## 3. Discussion

Oxidative stress is regarded as one of the triggers of endothelial dysfunction, and the imbalance between the overexpression of ROS and the defensive ability of cellular antioxidant systems is the main cause of endothelial damage. It has been evidenced that abnormal ROS production leads to unfavorable processes in cells, including damage to DNA, proteins, and lipids, which impairs the function of cells and, consequently, tissues. This, in turn, promotes the development and progression of cardiovascular disorders [5,6]. In our study, the H_2_O_2_ exposure was used as a model of oxidative stress in HBMVEC cells, and the H_2_O_2_ concentration was chosen based on the literature. It was found that H_2_O_2_ significantly decreased the cell viability and had an inhibitory effect on SOD, CAT, and GPx belonging to the antioxidant enzyme system, which effectively regulates the oxidation balance and inhibits the damage caused by oxidative stress. SOD is responsible for catalyzing the conversion of superoxide anions to H_2_O_2_. CAT and GPx peroxidases are involved in further transformation of hydrogen peroxide [22]. It was also noted that the H_2_O_2_ induction increased the MDA concentration, which is another marker of oxidative imbalance, and an increased MDA level indicates enhancement of lipid oxidation. Moreover, H_2_O_2_ lowered the NO level, an important factor regulating vasodilatation. Disrupted or abnormal expression of all these factors as a result of oxidative stress was described in the literature, and it is believed that they are involved in the pathogenesis of many diseases, including cardiovascular disorders [5,6]. In our work, diosmin and diosmetin were shown to have a protective effect on endothelial cells with induced oxidative stress via protection of the cells against the harmful effects of H_2_O_2_ and enhancement of the activity of cellular antioxidant systems. The DCFDA/H_2_DCFDA assay indicated that diosmin/diosmetin significantly alleviated H_2_O_2_-induced cytotoxicity. These compounds also strongly inhibited the H_2_O_2_-induced oxidative stress by influencing the enzyme system; the pretreatment with the flavonoids restored the CAT, SOD, and GPx activity in a concentration-dependent manner in the H_2_O_2_-treated cells. Interestingly, at the highest concentration, diosmetin also increased the SOD level in the control cells. It should also be mentioned that our previous research [18] showed minor direct scavenging activities in in vitro tests using DPPH and ABTS; however, this study demonstrated that both flavonoids, and especially diosmetin, exerted an antioxidative effect by enhancing the cellular antioxidant enzyme system activity. Moreover, the level of MDA was reduced in the H_2_O_2_-treated cells preincubated with the flavonoids, which suggests their protective effect against lipid peroxidation.

The impact of flavonoids on the activity of cellular enzymes has been reported in some papers; however, the results are ambiguous. Some researchers have described an increase [23,24,25], while others have reported no effect or even a decrease in the expression of antioxidant enzymes [26,27]. Contradictory results regarding the impact of flavonoid compounds on the antioxidant systems are results of strong structure–activity relationships, as described among others by Zhang et al [28].

Similar to our study, the activation of cellular antioxidant enzymes by flavonoid compounds in the H_2_O_2_-treated endothelial cells has been observed for quercetin [29], luteolin [30,31], and flavonoid-rich extract from the leaves of *Carya cathayensis* Sarg. [32] and from *Piper sarmentosum* Roxb. [33].

Oxidative stress also has a significant impact on vascular tone regulation. On the one hand, ROS favor vasoconstriction because they reduce the bioavailability of nitric oxide, which is a key factor in the regulation of vasodilation; on the other hand, H_2_O_2_ stimulates endothelial NO synthase (eNOS) and, therefore, the production of nitric oxide; thus, it shows a vasorelaxing effect. The disturbance in the NO level has a significant role in the development of cardiovascular disorders [6]. Sariya et al. suggested the vasodilator effects of diosmetin are relevant in reducing oxidative stress, raising endogenous antioxidant enzymes, and increasing nitric oxide bioavailability. In their study, the antioxidant effect of diosmetin was consistent with that of protein expression, such as Nrf2 and HO-1, leading to mitigated oxidative stress [34].

Our investigation shows that the H_2_O_2_ exposure decreased the level of endothelium-derived nitric oxide and diosmin/diosmetin upregulated its production. Some researchers suggest that the antioxidant effect may be a result of induced activation of the mitogen-activated protein kinase signaling pathway contributing to various processes, including the response to oxidative stress. MAPK proteins, such as p38, JNK, and ERK, are reported as important targets of H_2_O_2_. Moreover, the interaction between ROS and protein kinases is involved in the control of the vascular function. Abnormal levels of phosphorylated ERK, JNK, and p38 participate in the pathogenesis of cardiovascular disease [1,35].

The protective effect of diosmin and another flavonoid, hesperidin, against oxidative stress induced with acrylamide was previously evidenced in a rat model. For example, it was found that supplementation with flavonoids prevents lipid peroxidation and DNA damage induced by acrylamide. Moreover, pretreatment with diosmetin (but not diosmin) protected hepatocytes against oxidative stress caused by tert-butylhydroperoxide (TBHP), which was related to lower lipid peroxidation and higher glutathione content [20]. In turn, Mirzaee et al. found that diosmin had ameliorative effects on oxidative stress, significantly increased GSH levels, and restored catalase activity in paraquat-induced lung injury in mice [36].

Our findings demonstrate a protective effect of diosmin/diosmetin on H_2_O_2_-induced cell damage in the endothelium; however, it should be pointed out that diosmetin acted more effectively, probably as a result of partial blocking of the activity of diosmin by the sugar moiety. The higher activity of the aglycone form was also observed by Zaragozá et al., who investigated the inhibition of platelet aggregation and COX-1 [37] and the impact of these flavonoids on the level of pro-inflammatory factors in LPS-treated cells [38].

Our results showed a beneficial effect of the flavonoid in the alleviation of oxidative stress, which may be important in supporting the treatment of oxidative-stress-related disorders such as cardiovascular disease. However, it should also be mentioned that investigations based on cell lines could not be directly transferred into human studies because many other factors have to be considered; for example, the bioavailability and metabolism of the compounds. It should be noted that the maximal concentration of diosmin in plasma in CVD treatment is from several up to 50 ng/mL [19,39], which is a lower value than that used in our study. However, in experiments on cell lines, the concentrations used depend on cell sensitivity, and it has been shown that the flavonoids applied at lower concentrations exerted no effect on the endothelium treated with H_2_O_2_.

## 4. Materials and Methods

### 4.1. Cell Culture

The Human Brain Microvascular Endothelial Cells (HBMVEC) were obtained from iXCells Biotechnologies (San Diego, CA 92121, USA). The cells were cultured according to the standard protocol given by the manufacturer. The cells were grown in 96-well plates in CS-C medium (Sigma, St Louis, MO, USA) supplemented with 5% FBS (Sigma), 1% endothelial cell growth factor supplement (Sigma), and 1% penicillin solution (Sigma), and incubated at 37 °C in a humidified 5% CO2 incubator.

### 4.2. Experimental Design

The cells were seeded on the well bottom (1 × 10^5^ cells/mL) in 96-well plates. After 24 h of incubation at 37 °C, the cells were pretreated with the flavonoids (150–250 μM), and after 30 min, H_2_O_2_ (250 μM) was added to the medium to induce oxidative stress. After 6 h of cell stimulation, the NO (Nitric Oxide Assay Kit, ThermoFisher Scientific, Waltham, MA, USA), the MDA level, SOD, GSH-Px, and CAT (Abcam, Berlin, Germany) activities were determined using the kit according to the manufacturer’s instructions. Stock solutions of diosmin and diosmetin (Sigma) were prepared using DMSO/culture medium (1:1), and it was diluted. The final concentration of DMSO did not exceed 0.5%, and this concentration did not affect the cell viability.

### 4.3. Cytotoxicity Assay

#### 4.3.1. MTT Assay

The cells were treated with diosmin or diosmetin (100–500 μM), and after 24 h of incubation, 25 μL/well of a 3-(4,5-dimethylthiazole-2-yl)-2,5-diphenyltetrazolium bromide solution at a concentration 5 mg/mL (Sigma) was added, and the plates were incubated for 3 h at 37 °C. The crystals were solubilized in 10% sodium dodecyl sulfate (SDS) in 0.01M HCl, and absorbance was measured at λ = 570 nm wavelength with the use of an E-max Microplate Reader (Molecular Devices Corporation, Menlo Park, CA, USA). To establish cytotoxicity in H_2_O_2_-treated cells, the cells were pretreated with the flavonoids (150–250 μM), and after 30 min, H_2_O_2_ (250 μM) was added to the medium. MTT assay was carried out after 6 h of cell stimulation.

#### 4.3.2. Alamar Blue Assay

The cells were treated with diosmin or diosmetin (100–500 μM), and after 24 h of incubation, a Resazurin solution (60 μM) was added. The plates were incubated for 2 h at 37 °C. Then, fluorescence was measured at λ = 570 nm wavelength using a FilterMax F5 microplate reader (Thermo Fisher Scientific). To establish cytotoxicity in H_2_O_2_-treated cells, the cells were pretreated with the flavonoids (150–250 μM), and after 30 min, H_2_O_2_ (250 μM) was added to the medium. AB assay was carried out after 6 h of cell stimulation.

### 4.4. Quantification of Intracellular Reactive Oxygen Species

The generation of intracellular reactive oxygen species (ROS) in the endothelial cells was performed as previously described [40]. After 24 h of incubation, the medium was removed and replaced with 10 μM H_2_DCFDA (Sigma Aldrich), and the cells were incubated for 45 min at 37 °C. The fluorescence was measured after 90 min using a FilterMax F5 microplate reader (Thermo Fisher Scientific) at a maximum excitation of 485 nm and emission spectra of 530 nm. The concentrations of flavonoids were 150, 200, and 250 μM.

### 4.5. Statistical Analysis

The results are means ± SD of three independent measurements (*n* = 3). The data were analyzed using one-way ANOVA followed by Dunnett’s multiple comparison post hoc test. Additionally, Student’s test was performed to assess the differences between flavonoid concentrations. Differences were considered significant at *p* < 0.05. All statistical analyses were performed using Statistic ver. 13.3 software (Tibco Software Inc., Palo Alto, CA, USA).

## 5. Conclusions

Our study has shown that diosmin and diosmetin may protect endothelial cells against oxidative stress caused by H_2_O_2_ exposure, as they alleviate the cytotoxicity via an impact on the cellular antioxidant enzymatic system. It has been demonstrated that the protective effects were strongly associated with the increased activity of CAT, GPx, and SOD, i.e., enzymes responsible for cellular redox homeostasis. Moreover, they restored the NO level. Based on these findings, it can be supposed that flavonoids may support the treatment in oxidative stress-related disorders.

## Data Availability

The data presented in this study are available on request from the corresponding author.

## Supplementary Materials

The following supporting information can be downloaded at: https://www.mdpi.com/article/10.3390/molecules27238232/s1, Figure S1: Cell viability determined by the MTT (a) and Alamar Blue (b) assay expressed as a percentage of control (0.5% DMSO). Cells were treated with diosmin/diosmetin at different concentrations. The data are means ± SD (*n* = 3). One-way ANOVA followed by Dunnett’s multiple comparison post hoc test; *indicates statistically significant difference (*p* < 0.05) versus untreated controls; Figure S2: May-Grünwald–Giemsa (MGG) staining of endothelial cells: (a) control, (b) cells treated with H_2_O_2_, (c) cells preincubated with diosmin prior to H_2_O_2_ exposure, (d) cells preincubated with diosmetin prior to H_2_O_2_ exposure. Magnification 200×. Bar = 20 μm; Figure S3: Intracellular Reactive Oxygen Species (H_2_DCFDA assay): (a) control, (b) cells treated with H_2_O_2_, (c) cells preincubated with diosmin prior to H_2_O_2_ exposure, (d) cells preincubated with diosmetin prior to H_2_O_2_ exposure; Figure S4: Effect of the diosmin/diosmetin treatment and the H_2_O_2_ exposure on the antioxidant enzyme activity calculated as a percentage in comparison with the untreated control. (a)—relative activity of superoxide dismutase (SOD), (b)—relative activity of catalase (CAT) (c)—relative activity of glutathione peroxidase (GPx) (d)—relative malondialdehyde (MDA) concentration. The data are means ± SD (*n* = 3). * indicates a statistically significant difference (*p*<0.05) versus the control cells. One-way ANOVA followed by Dunnett’s multiple comparison post hoc test; Figure S5: Effect of the diosmin/diosmetin treatment and the H_2_O_2_ exposure on the NO level calculated as a percentage in comparison with the untreated control. The data are means ± SD (*n* = 3). *indicates a statistically significant difference (*p* < 0.05) versus the control cells. One-way ANOVA followed by Dunnett’s multiple comparison post hoc test.
