# Supplementary material for: Antioxidant Potential of Diosmin and Diosmetin against Oxidative Stress in Endothelial Cells
Source: Molecules. 2022 Nov 25;27(23):8232. doi: 10.3390/molecules27238232 (PMC9739697; doi:10.3390/molecules27238232)
Supplement: Supplementary file 1 [file molecules-27-08232-s001.zip › molecules-2029066-supplementary.pdf]

molecules

MDPI

Supplementary material

Antioxidant Potential of Diosmin and Diosmetin against Oxidative Stress in Endothelial Cells

Magdalena Wójciak [1], Marcin Feldo [2], Grzegorz Borowski [2], Tomasz Kubrak [3], Bartosz J. Płachno [4] and Ireneusz Sowa [1,*]

[1] Department of Analytical Chemistry, Medical University of Lublin, Lublin, Chodzki 4A, 20-093, Poland
[2] Department of Vascular Surgery, Medical University of Lublin, Staszica 11 St., 20-081 Lublin, Poland
[3] Department of Biochemistry and General Chemistry, Medical College of The University of Rzeszów, Rzeszów, 2A Kopisto St., 35-959 Rzeszów, Poland
[4] Department of Plant Cytology and Embryology, Institute of Botany, Faculty of Biology, Jagiellonian, University in Kraków, Gronostajowa 9 St. 30-387 Cracow, Poland
* Correspondence: i.sowa@umlub.pl; Tel.: +48817185551

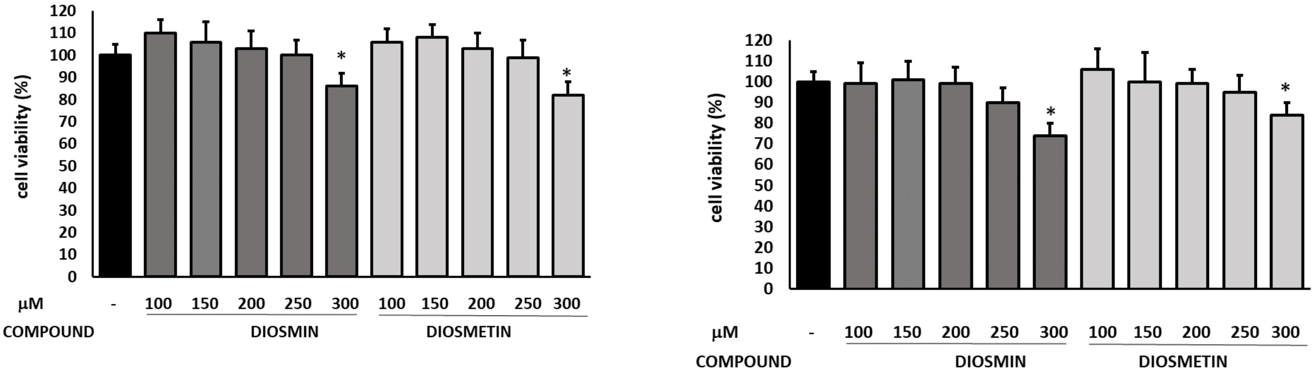

(a) (b)

Figure S1. Cell viability determined by the MTT (a) and Alamar Blue (b) assay expressed as a percentage of control (0.5% DMSO). Cells were treated with diosmin/diosmetin at different concentrations. The data are means ± SD (n = 3). One-way ANOVA followed by Dunnett's multiple comparison post hoc test; *indicates statistically significant difference ($p < 0.05$) versus untreated controls.

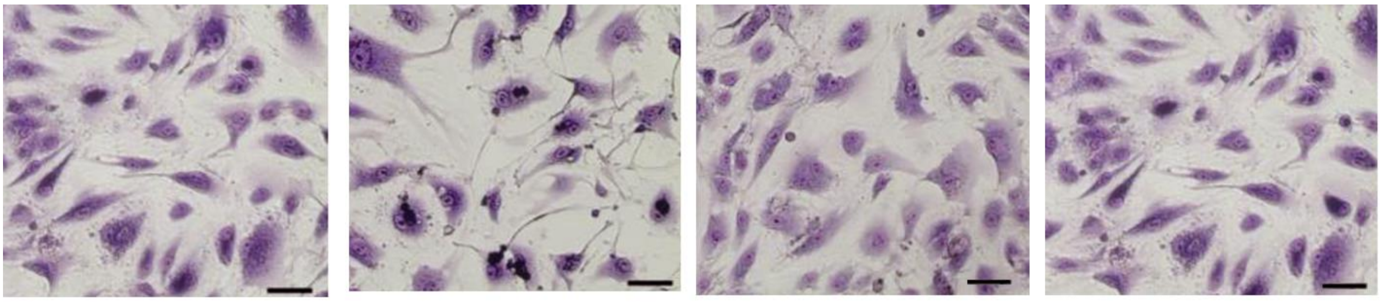

(a) (b) (c) (d)

Figure S2. May-Grünwald–Giemsa (MGG) staining of endothelial cells: (a) control, (b) cells treated with H_2O_2, (c) cells preincubated with diosmin prior to H_2O_2 exposure, (d) cells preincubated with diosmetin prior to H_2O_2 exposure. Magnification 200×. Bar = 20 µm.

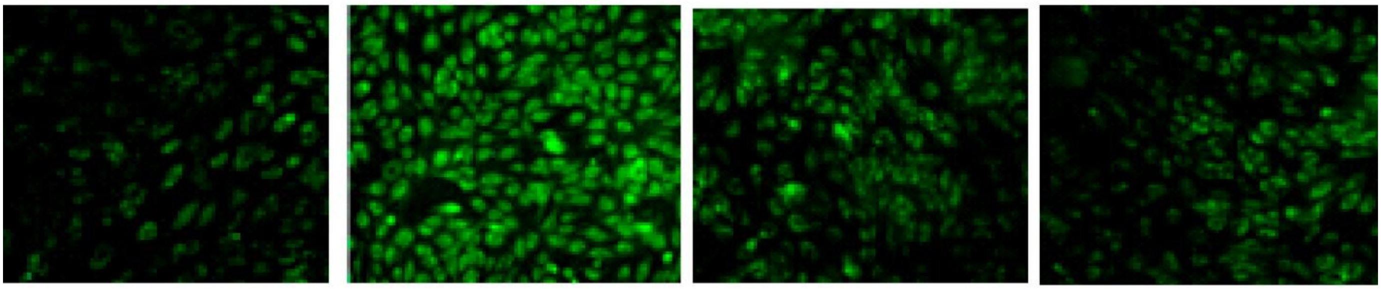

(a) (b) (c) (d)

Figure S3. Intracellular Reactive Oxygen Species (H2DCFDA assay): (a) control, (b) cells treated with H_2O_2, (c) cells preincubated with diosmin prior to H_2O_2 exposure, (d) cells preincubated with diosmetin prior to H_2O_2 exposure.

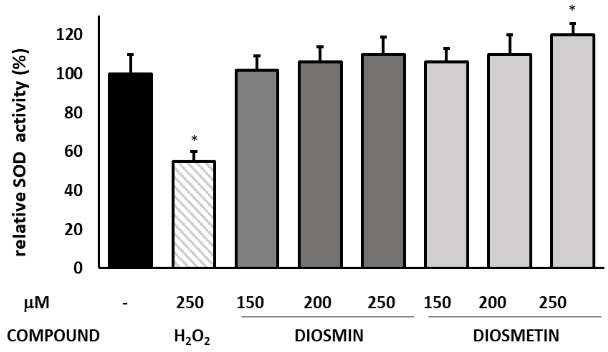

(a)

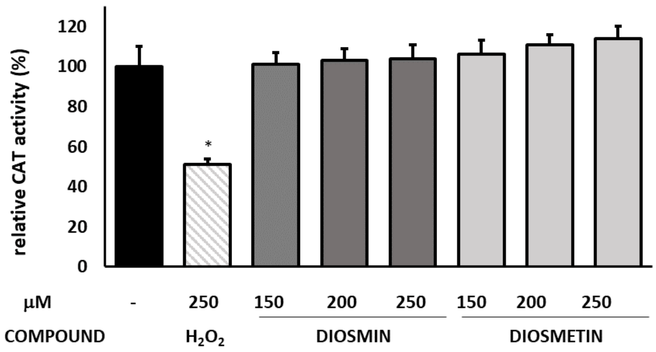

(b)

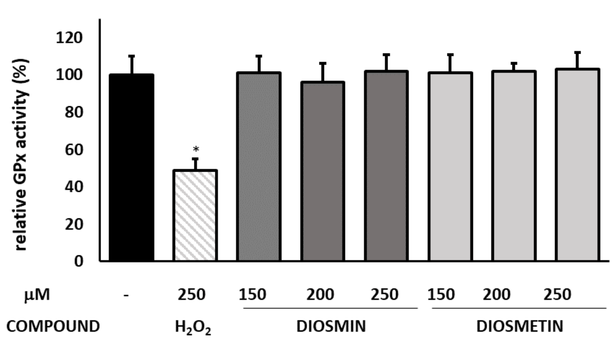

(c)

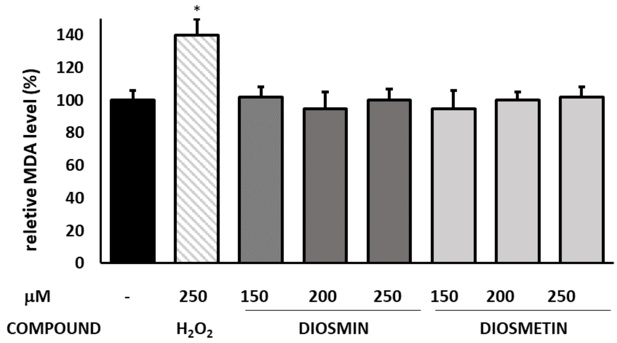

(d)

Figure S4. Effect of the diosmin/diosmetin treatment and the H_2O_2 exposure on the antioxidant enzyme activity calculated as a percentage in comparison with the untreated control. (a)–relative activity of superoxide dismutase (SOD), (b)–relative activity of catalase (CAT) (c)–relative activity of glutathione peroxidase (GPx) (d)–relative malondialdehyde (MDA) concentration. The data are means ± SD (n = 3). * indicates a statistically significant difference ($p<0.05$) versus the control cells. One-way ANOVA followed by Dunnett's multiple comparison post hoc test.

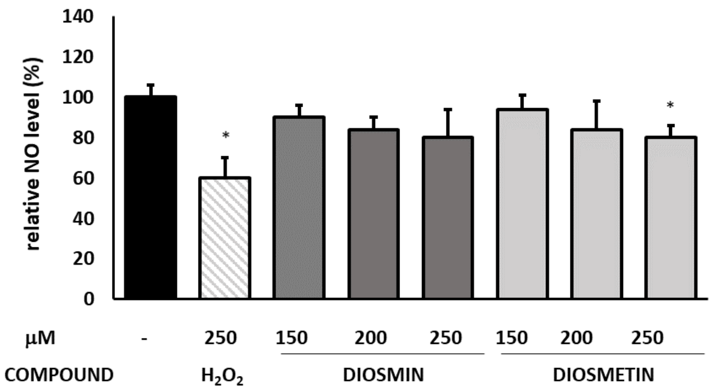

Figure S5. Effect of the diosmin/diosmetin treatment and the H_2O_2 exposure on the NO level calculated as a percentage in comparison with the untreated control. The data are means ± SD (n = 3). *indicates a statistically significant difference ($p < 0.05$) versus the control cells. One-way ANOVA followed by Dunnett's multiple comparison post hoc test.
